# A computational study of red blood cell deformability effect on hemodynamic alteration in capillary vessel networks

**DOI:** 10.1038/s41598-022-08357-z

**Published:** 2022-03-11

**Authors:** Saman Ebrahimi, Prosenjit Bagchi

**Affiliations:** Mechanical and Aerospace Engineering Department, Rutgers, The State University of New Jersey, Piscataway, NJ 08854 USA

**Keywords:** Engineering, Biomedical engineering, Blood flow

## Abstract

Capillary blood vessels, the smallest vessels in the body, form an intricate network with constantly bifurcating, merging and winding vessels. Red blood cells (RBCs) must navigate through such complex microvascular networks in order to maintain tissue perfusion and oxygenation. Normal, healthy RBCs are extremely deformable and able to easily flow through narrow vessels. However, RBC deformability is reduced in many pathological conditions and during blood storage. The influence of reduced cell deformability on microvascular hemodynamics is not well established. Here we use a high-fidelity, 3D computational model of blood flow that retains exact geometric details of physiologically realistic microvascular networks, and deformation of every one of nearly a thousand RBCs flowing through the networks. We predict that reduced RBC deformability alters RBC trafficking with significant and heterogeneous changes in hematocrit. We quantify such changes along with RBC partitioning and lingering at vascular bifurcations, perfusion and vascular resistance, and wall shear stress. We elucidate the cellular-scale mechanisms that cause such changes. We show that such changes arise primarily due to the altered RBC dynamics at vascular bifurcations, as well as cross-stream migration. Less deformable cells tend to linger less at majority of bifurcations increasing the fraction of RBCs entering the higher flow branches. Changes in vascular resistance also seen to be heterogeneous and correlate with hematocrit changes. Furthermore, alteration in RBC dynamics is shown to cause localized changes in wall shear stress within vessels and near vascular bifurcations. Such heterogeneous and focal changes in hemodynamics may be the cause of morphological abnormalities in capillary vessel networks as observed in several diseases.

## Introduction

Microvascular networks in human body are made of the smallest blood vessels, namely, capillaries, arterioles and venules. The architecture of a microvascular network is complex and characterized by constantly bifurcating and merging vessels. The topology of networks can vary from organ to organ and differ under healthy and disease conditions^1–7^. Moreover, these vessels are not necessarily straight, and often highly winding. The primary function of the microvascular network is to carry out the delivery of oxygen and other metabolites to tissues, and clearance of tissue waste. Red blood cells (RBC), which constitute nearly 45% of blood volume, serve as the oxygen carrier. RBCs also facilitate regulation of blood flow in the microcirculation by releasing adenosine triphosphate (ATP) which then initiates production of nitric oxide (NO), a vasodilator, by endothelial cells (EC). They also mediate NO bioavailability, acting as both a scavenger of vascular NO, and as a source of NO bound to hemoglobin^8,9^.

An individual RBC is made of hemoglobin, which acts like a viscous fluid, and is encapsulated by a surrounding viscoelastic membrane that is highly flexible^10,11^. Under healthy conditions, a human RBC assumes a resting shape of a biconcave discoid of about 7.5–8 μm diameter. Flexibility of the membrane and fluidic nature of hemoglobin make RBCs extremely deformable. They can easily squeeze and flow through capillary vessels of significantly smaller diameter. RBC deformability, hematocrit, and cell-to-vessel diameter ratio, among other factors, are responsible for the diameter-dependence of apparent viscosity of blood, often referred to as the Fahraeus-Lindqvist effect^12,13^. Under healthy conditions, arrangement of a microvascular network is optimized to maintain tissue perfusion and, hence, oxygen delivery. Yet, in vivo studies have shown that the distribution of RBCs in a network is both spatially and temporally heterogeneous^14–17^. While many factors contribute to this heterogeneity, including dilation of individual vessel and capillary plugging by leukocytes, recent studies have shown that cellular-scale phenomena also dictate RBC distribution in the network. These include, among others, the lingering behavior of an individual RBC at a capillary bifurcation where the cell can stretch significantly and reside near the stagnation-point longer than a freely flowing cell^18–23^, and a radially skewed hematocrit profile over a vessel cross-section caused by the presence of upstream bifurcations^14,17,22–26^.

Many pathological conditions, such as sickle cell disease, malaria, diabetes mellitus, and sepsis, are associated with a loss of RBC deformability^27–34^. Cell deformability is also reduced during storage of RBC, in post-ischemic reperfusion and in acute inflammation^35–38^. Consequently, blood viscosity increases, and shear-thinning behavior is reduced as shown by in vitro viscometry and microfluidic studies, as well as computer modeling^39–43^. Because of the pathophysiological importance, many studies in the past were carried out in vivo that addressed the influence of RBC deformability on microvascular hemodynamics using chemically treated cells, diseased cells and stored cells. These studies have shown that less deformable RBCs can affect blood flow in multiple ways. Increased viscosity due to reduced deformability causes a higher vascular resistance and, hence, reduced tissue perfusion and altered oxygen delivery^36,43–48^. Wall shear stress, which affects EC functioning, is also affected by RBC deformability^36,46^. Furthermore, spatial distribution of cells across the networks was observed to become more heterogeneous with decreasing deformability^30,43,49^. Less deformable cells tend to flow through vessels of less resistance resulting in a decrease in functional capillary density and capillary recruitment in response to tissue hypoxia^47,50^. Increased capillary transit time and vessel occlusion are also reported in presence of such cells^30,49–53^. In addition to such direct hemodynamic impact, RBC deformability can also affect flow regulation^8,9,54^. Since ATP release is dependent on RBC deformation, the lack of ATP release can lead to a reduced NO production and, consequently, hindered vasodilation^8,9,46,54,55^. Reduced deformability also affects oxygen release from RBCs. For example, diabetic RBCs have a higher O_2_ affinity and less O_2_ release compared to healthy RBCs^56^. Hypoxia-mediated NO release from RBC-bound hemoglobin was shown to diminish under hyperglycemia^57,58^.

Concomitant with reduced RBC deformability, pathological conditions such as diabetic retinopathy and nephropathy, and sickle cell vasculopathy, are marked by visible morphological changes in capillary vessels^3–5,59,60^. These changes include vessel regression, increased vessel tortuosity, and appearance of microaneurysms. These abnormalities appear to be spatially heterogeneous and ‘focal’. In clinical settings, they are used to determine the onset, progression, and severity of diseases. However, hemodynamic mechanisms, if any, underlying these changes remain largely unknown. We hypothesize that reduced RBC deformability causes spatially heterogeneous and focal changes in capillary hemodynamics, which eventually trigger vascular abnormalities that are also heterogeneous and focal. Although it is now clearly recognized that capillary perfusion may be attenuated because of diminished RBC deformability, it is generally unknown whether hemodynamic changes are focal and heterogeneous. Furthermore, the cellular-scale mechanisms, such as RBC flow behavior at vascular bifurcations, that may cause such focal and heterogeneous changes have not also been elucidated.

The objective of this study is, therefore, to quantify hemodynamic alterations in microvascular networks caused by reduced RBC deformability. To that end, we undertake a computational modeling approach simulating flow of normal and less deformable RBCs through in silico microvascular networks^22^. Previous models of capillary network hemodynamics have treated vessels in microvascular networks as 1D straight segments neglecting the geometric complexity and details^61,62^. Such models also do not consider the deformation and flow of individual red blood cell, and instead use empirical relations to prescribe blood viscosity, as well as cell distribution at a vascular bifurcation. In contrast, the model used in this study is fully 3D and it retains exact geometric details of physiologically realistic microvascular networks, and deformation of every RBC as they flow through the vessels. Fully resolved computational models have been used in recent years to study RBC flow dynamics at vessel bifurcations. Many of such studies, however, considered single bifurcations in isolation^20,63,64^. In contrast, the current model considers many bifurcations in sequence as they appear in vivo, as well as vessel tortuosity, allowing a *natural* development of RBC distribution across the simulated networks resembling physiological distribution. Using this model, we quantify changes in RBC partitioning, heterogeneity in RBC distribution, perfusion and vascular resistance, and wall shear stress due to reduced deformability, and elucidate the cellular-scale mechanisms that cause such changes to appear in a spatially heterogeneous and focal manner.

## Methodology

**Figure 1:**
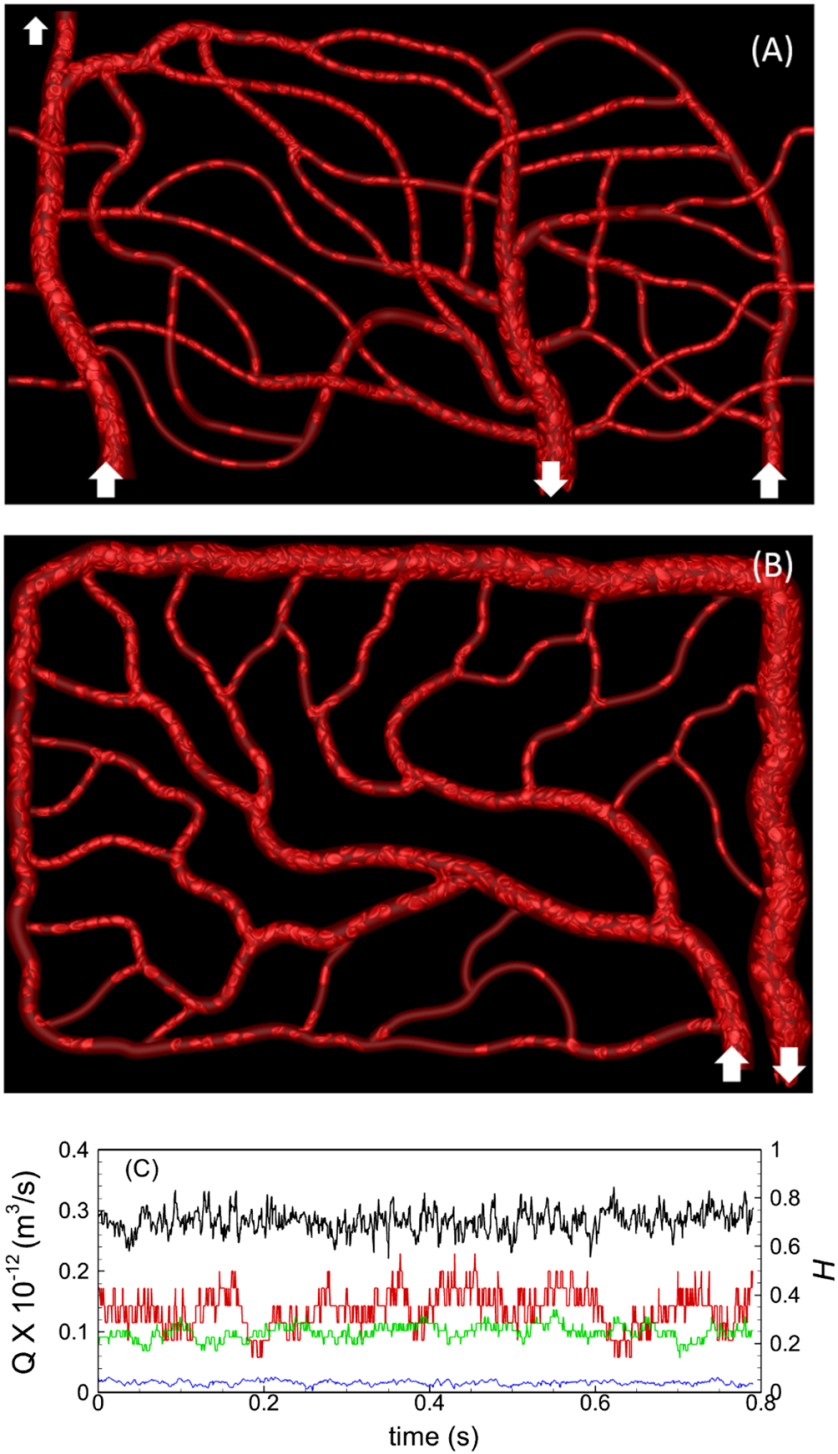
(**A**,**B**) Representative instantaneous visualizations showing RBC flow and distribution in networks A and B. Arrows indicate inlets/outlets. (**C**) Flow rates ($Q$, black, blue) and hematocrits ($H$, red, green) in two representative vessels are shown to indicate the flow reached a quasi-steady state.

The computational methodology and simulation details are given in our previous publications^22,23,65^. We have used this methodology to predict spatial and tempral heterogeneity in RBC distribution, RBC partitioning in bifurcations, three-dimensionality of cell-depelted layer, WSS, and WSS-gradient in physiologically realistic microvascular networks^66,67^. We extend this method here to two new networks as shown in Fig. 1 that were developed following in vivo images. Specifically, network A (Fig. 1A) mimicks the capillary networks in a rectangular area near the foveal avascular zone in the human retina. The corresponding in vivo image is given in^68^. This region is the most visually acute region, and yet it is known to be greatly affected in diabetic retinopathy with the appearance of microaneurysms, increased vessel totuoisity and capillary vessel regression, and is, therefore, pathophyiologically significant. Network B (Fig. 1B) resembles an area in rat mesenteric vasculature^69^. It should be noted that some geometric modifications, such as altering the inlets and outlets, to the in vivo images are needed in order to make them computaionally amenable and efficient. As evident, the in silico networks comprised of multiple bifurcations, mergers, and winding vessels. Multiple inlets and outlets can be present in the model; in addition, vessels need not lie in the same plane, as can be seen in Fig. 1A. Vessel diameters range from 5.5 to 24 μm. Projected tissue areas simulated are about $450\times 300$ µm^2^.

The numerical methodology is based on the immersed-boundary methods^65^. The in silico networks are first built using a CAD software. The centerline geometry is created in 3D CAD modeling using features such as Spline, Line, Arc, etc. Geometric constraints are then added to achieve desired structures. Three-dimensionality is added to the cross-sections by extruding along the centerlines and connecting with other vessels at bifurcation and merger regions. Finally, smoothness around the geometry at bifurcations and mergers is added. Vessel walls are discretized with a triangulated surface mesh either using the surface meshing functionality of the CAD software, or the open-source mesh generator Gmsh.

The network is then imported to the simulation code. A rectangular box is defined as the computational domain that encloses the network. The domain is discretized with rectangular Cartesian mesh of approximately 160 million points. As is the case for immersed-boundary methods, the governing equations of the fluid motion, which are the continuity and unsteady Stokes equations for the present study, are solved in the entire box. The surface mesh on the vessel walls separates the vessel interior (lumen), which is the region of interest, from the exterior. Vessels are assumed to be nondeformable, and the no-slip condition is implemented at the wall using the sharp-interface ghost-node method. A projection method is used to obtain the fluid velocity and pressure in conjunction with a staggered-grid finite-volume/spectral approach.

Each RBC is modeled as a sack of fluid enclosed by a zero-thickness hyperelastic membrane, and having a biconcave discocyte resting shape with an end-to-end distance of 7.8 μm^10,11^. The RBC membrane is assumed to possess a resistance against shearing, area dilation, and bending. The shearing deformation and area dilation are modeled using the strain energy function developed by Skalak et al.^70^, and the bending resistance is modeled following Helfrich^71^. The viscosity of the hemoglobin and plasma is taken to be 0.006 and 0.0012 Pa-s, respectively^11^. Full 3D deformation of the cells is predicted and coupled to the fluid flow via the immersed-boundary method. The governing flow equations are solved both inside and outside the cells, taking into consideration the viscosity difference of two fluids. Each RBC surface is discretized using 5120 triangular elements, and a finite-element method is used to compute the elastic stresses in the membrane. Multiple parameters determining both morphological and mechanical properties of RBC may change during disease or storage. Here we consider one property, namely, the 2D shear elastic modulus ${G}_{S}$ of the cell membrane. For ‘*normal*’ healthy cells, we take ${G}_{S}=5\times {10}^{-6}$ N/m^10,11^. For less deformable cells, hereafter termed ‘*stiffer*’ cells, we assume a 10 times higher value, which agrees with a recent experimental measurement of elastic modulus of stored RBCs^35^. Other parameters, namely, the cell volume and resting cell shape, hemoglobin viscosity, and membrane bending modulus are taken to be identical for normal and stiffer cells. It should be noted that our focus is not to address hemodynamics under any specific disease condition or stored RBCs; rather we consider a generic study by increasing the RBC membrane elastic modulus as a means of reducing the cell deformability.

Blood flow in the model networks is driven by specifying either a pressure or flow rate boundary condition. In total, six simulations are performed: Simulations with the pressure boundary condition for network A where a physiological pressure difference of 4.8 mmHg is specified between the inlet and outlet; simulations with the flow rate boundary condition for networks A and B with a physiological flow rate of 1.2–1.3 nL specified in the main feeding artery^10^. For each of these cases, simulations are performed for normal cells and stiffer cells. RBCs are injected at the feeding artery with an average sustained hematocrit of 30%. They are naturally distributed by the flow throughout the networks. At any instant, network A contains about 750 RBCs, and network B about 1150 RBCs. The number of RBCs is kept nearly the same for normal and stiffer cells. Hemodynamic quantities reach a quasi-steady state after the initial 0.2–0.4 s of physical flow time, after which the data collection is performed for about 0.8–1 s. The 3D velocity and pressure fields are stored every 5 ms, and the cell shapes are stored at every 0.5 ms. Resulting average blood velocity in the feeding arteriole is about 5 mm/s and predicted velocities in capillary vessels range $\sim$ 0.7–3 mm/s, in agreement with in vivo data^10,72,73^. A sample flow rate and hematocrit versus time plot for two vessels in a network is given in Fig. 1c, to show that hemodynamic quantities are converged to a quasi-steady state.

It may be noted that our simulation technique and the method of analysis are similar to our previous studies^22,23,66,67^. However, in those studies we did not address the specific question of the effect of reduced RBC deformability on network-scale hemodynamics. Also, the in silico networks used here are new, and geometrically (and numerically) more complex than those considered previously.

Quantitative validation for network hemodynamic parameters for normal RBCs has been presented in our previous studies^22,66^. Additional comparison against in vivo or in vitro studies is noted in later sections wherever possible.

## Results

Visualizations of instantaneous RBC distribution from the two networks are presented in Fig. 1. Large deformation of RBCs in capillary vessels, and a wide range of shapes and flow patterns as observed in vivo can also be observed in the simulations. These include bullet/parachute and slipper shapes, and single-file flow in capillary vessels and multi-file flow in larger vessels. Spatially heterogeneous distribution of RBCs which is a hallmark of microvascular blood flow in vivo is also observed across each simulated network as some capillary vessels are seen to be filled with cells, while some vessels are sparsely populated. Despite a reduction in cell deformability, we did not observe a complete flow blockage in any capillary vessel.

**Figure 2:**
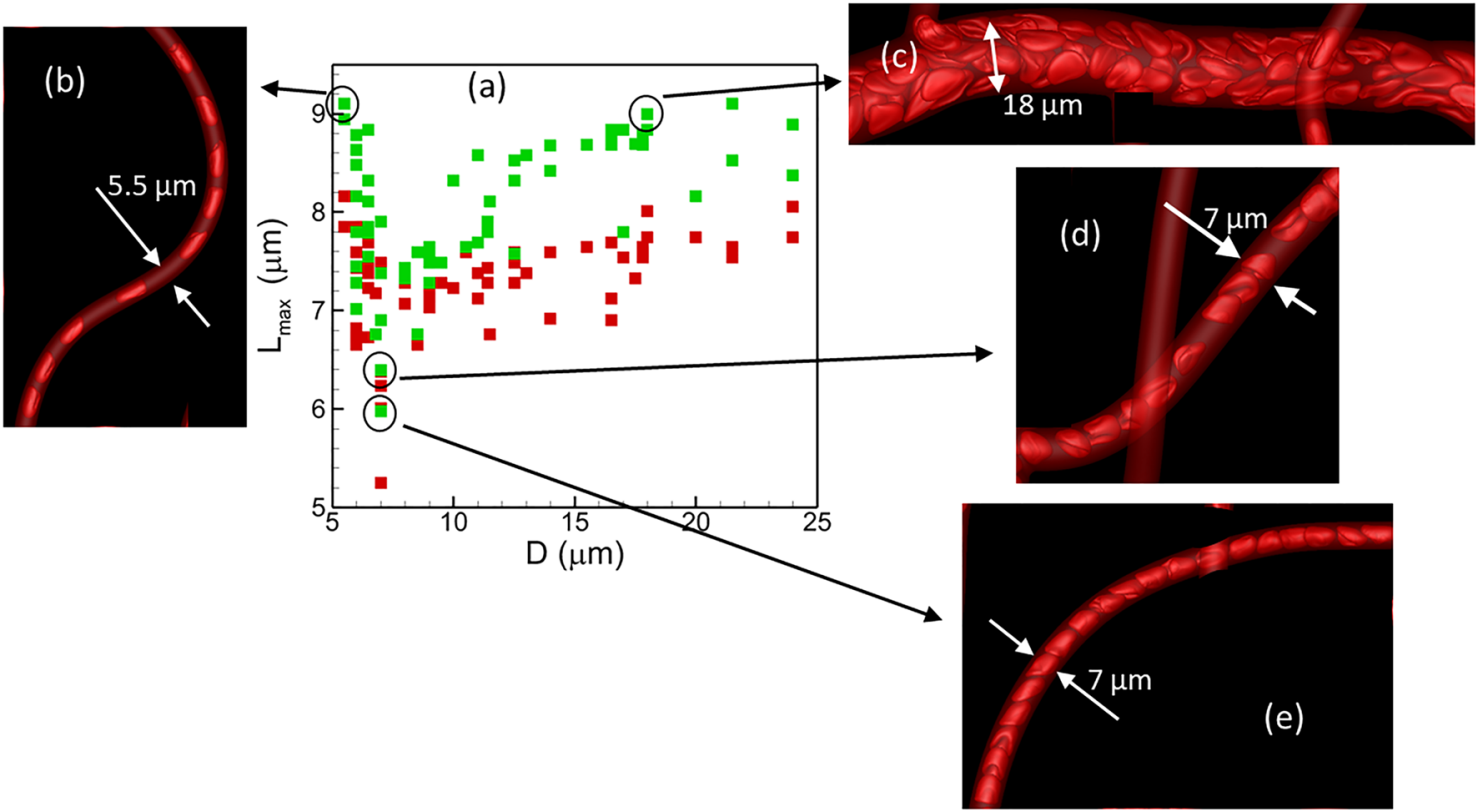
(**a**) Average cell length in each vessel as a function of vessel diameter for normal (green) and stiffer (red) cells. (**b**–**e**) show cell shape in vessels of different diameters. Vessels in (**d**,**e**) have same diameter but different hematocrit, 0.22 and 0.37, respectively.

Average cell length in each vessel is shown in Fig. 2a. Cell length is computed as the maximum end-to-end distance. In all vessels, average cell length is reduced as a result of reduced deformability. Interestingly, a non-monotonic trend of the cell length with respect to vessel diameter is predicted for both healthy and stiffer cells. Average lengths are higher in vessels of diameter $\lesssim$ 6 μm and $\gtrsim 12\upmu$m. The minimum cell lengths are predicted in diameter range $\sim$ 7–11 μm. For vessel diameter less than $\sim$ 7 μm, cell length increases with decreasing diameter, and vice versa in the range $\gtrsim 12\upmu$m. Figure 2 also shows deformed cells shapes in example vessels of the networks to explain such trends. In vessels less than $\sim$ 6 μm diameter, cells are significantly extended due to the geometric confinement resulting in higher length (Fig. 2b). In vessels of diameter $\gtrsim 12\upmu$m, cells flow in double or multi-file manner. This causes the ones flowing near the vessel wall to be stretched due to locally higher shear rate, resulting in higher end-to-end cell length (Fig. 2c). In the intermediate range ($\sim$ 7–11 μm diameter), cell elongation is less because of reduced confinement and single-file flow (Fig. 2d). Additionally, at a higher hematocrit in such vessels, cells flow “back-to-back”, causing a reduction in cell length (Fig. 2e). Despite the general trend as described, a strong heterogeneity in cell length in vessels of similar caliber can be noted. One reason for heterogeneity in cell length is a heterogeneity in hematocrit and flow rate in vessels of similar caliber as discussed below.

### RBC distribution and partitioning

**Figure 3:**
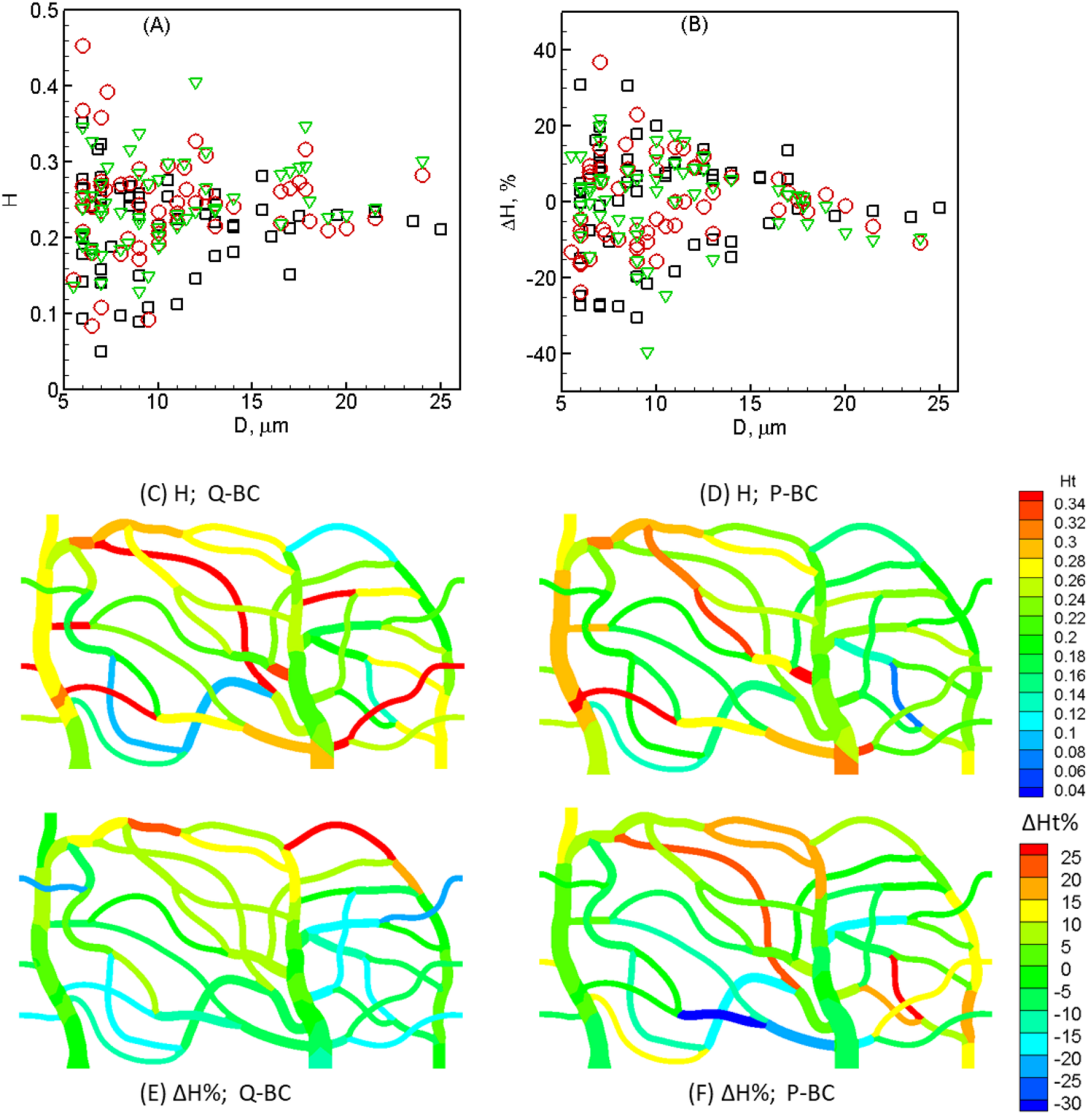
(**A**) Time-averaged hematocrit $H$ for normal cells in each vessel of both networks and for all boundary conditions (O—network A, flow-rate condition; $\nabla$—network A, pressure condition; $\square$—network B, flow-rate condition). (**B**) Hematocrit change $\Delta H$ in each vessel caused by reduced deformability. (**C**,**D**) Map of $H$ in network A for flow-rate (Q) and pressure (P) boundary conditions. (**E**,**F**) Map of $\Delta H$.

Figure 3A shows time-averaged hematocrit $H$ predicted for each vessel in the two networks and for all boundary conditions for normal RBCs. As seen, $H$ varies from less than 0.1 to above 0.4, with the mean about 0.21–0.24, as is the case in vivo, where average hematocrit is known to be less than systematic hematocrit^10,14–17,24^. A high degree of spatial heterogeneity is noted here, with vessels of same caliber having very different hematocrits. Maximum degree of heterogeneity is observed for terminal capillaries. Spatial maps of $H$ in network A for the flow rate and pressure boundary conditions are shown in Fig. 3C,D, respectively. Geographic heterogeneity of $H$ is evident here which arises due to the natural distribution of cells in our simulations, as is the case in vivo.

To quantify the effect of RBC deformability on RBC distribution across the networks, we compute percentage change of time-averaged hematocrit in each vessel as $\Delta H=({H}_{stiffer}-{H}_{normal})/{H}_{normal}$ ×100, and present in Fig. 3B. A range of ± 40% is predicted for $\Delta H$ with the terminal capillaries showing the highest changes. Positive and negative values indicate, respectively, that while $H$ increases in some vessels, it decreases in others. A high degree of heterogeneity is also seen in $\Delta H$, with vessels of same caliber showing different degree of changes. Maps of $\Delta H$ are given in Fig. 3E,F for network A, which shows that the changes are not localized to specific geographic regions. Note that the spatially averaged $\Delta H$ in our simulations is nearly zero, since the total numbers of RBCs are nearly the same for both normal and stiffer cells. Therefore, a network-wide increase/decrease of $H$ is not predicted, but a significant change at the level of individual vessel is predicted. Coefficient of variation of $H$ across all vessels is 0.26–0.32 for normal cells and 0.29–0.39 for stiffer cells, depending on the specific network and boundary condition. Thus, despite several individual vessels showing a significant change in $H$ as a result of reduced RBC deformability, heterogeneity of $H$ in the entire networks in presence of stiffer cells increase by a smaller degree compared to normal cells. These results indicate that despite the total number of RBCs and the feeding hematocrit being fixed in our simulations, stiffer RBCs take different pathways as compared to normal RBCs.

We now investigate the cellular-scale mechanisms that cause large hematocrit changes in individual vessels as predicted above. For blood vessels in a network, hematocrit is determined by how cells are distributed into the downstream (daughter) vessels as they flow through a bifurcation. This is referred to as cell partitioning, and for a bifurcation it is primarily dictated by the ratio ${Q}^{*}$ of the flow rate in a daughter vessel to that in the mother (upstream) vessel^10,12,14,17–26^. For vessels of diameter comparable to the size of an individual RBC, the partitioning in general is not proportional to the flow rates; rather it is disproportional, with the daughter vessel having the higher flow rate ratio getting even a higher fraction of RBCs. Previous studies and our own modeling have shown that while this is generally the case, under certain conditions however the opposite behavior can be observed in which the daughter vessel with the higher flow fraction can get a lower fraction of cells^14,19–23^. Accordingly, the former is termed as *regular* partitioning and the latter as *reverse* partitioning.

**Figure 4:**
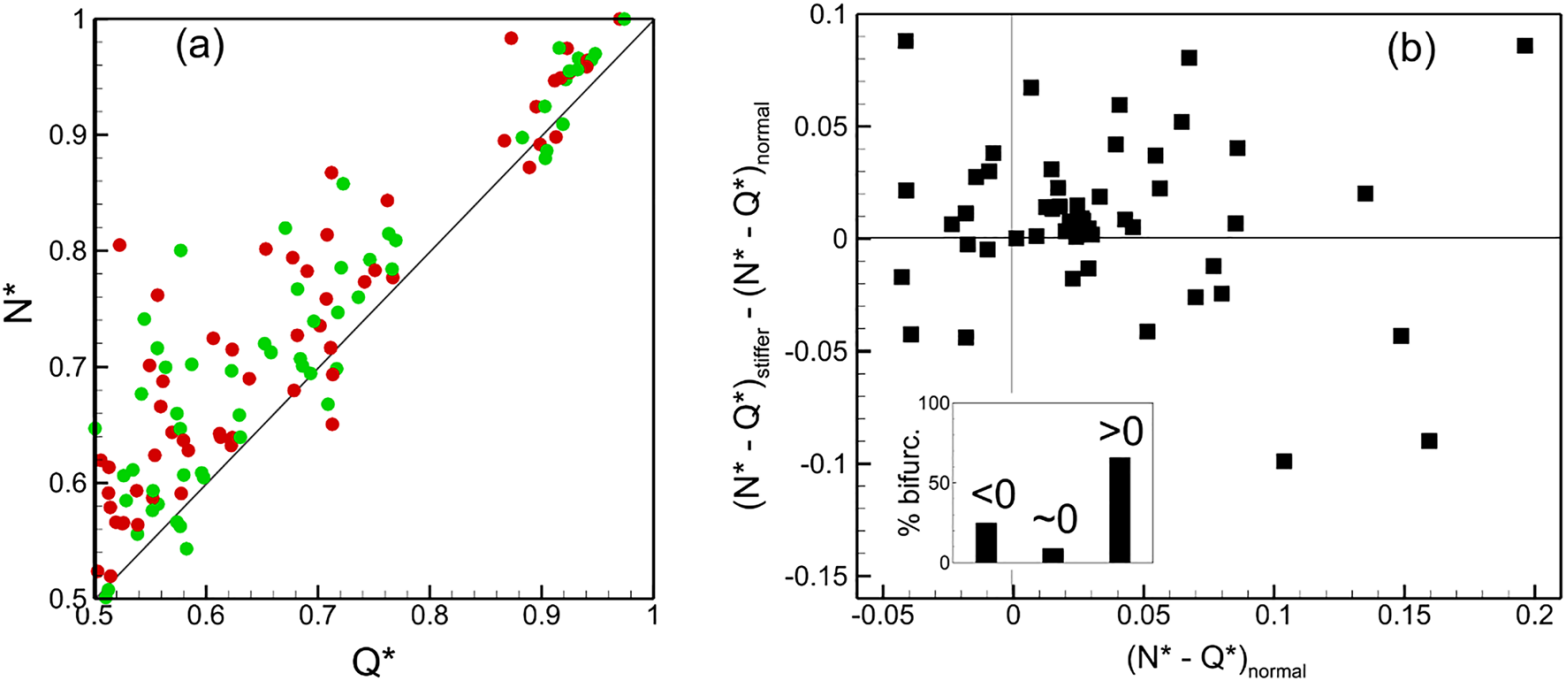
(**a**) RBC partitioning at each bifurcation in the two networks for both boundary conditions. Green and red circles represent normal and stiffer cells, respectively. (**b**) Change in RBC partitioning defined as ${\Delta }_{N-Q}={({N}^{*}-{Q}^{*})}_{stiffer}-{({N}^{*}-{Q}^{*})}_{normal}$. Inset shows % of total bifurcations for which ${\Delta }_{N-Q}<0, \sim 0,$ or $>0$.

Figure 4a shows the present results for time-averaged cell partitioning in the two networks, for both normal and stiffer cells. Data is presented in terms of RBC flux ratio ${N}^{*}$, which is the ratio of the RBC flux in a daughter vessel to that in the mother vessel at a bifurcation, versus the flow rate ratio ${Q}^{*}$. Note that our simulations predict time-dependent RBC flux ratio and flow rate ratio, from which the time averages are obtained. Each data point in the plot represents an individual bifurcation in the two networks for each boundary condition simulated. As seen for both cell types, while most bifurcations show the regular partitioning (${N}^{*}>{Q}^{*}$, if ${Q}^{*}>0.5$), a small number of them shows the reverse partitioning (${N}^{*}<{Q}^{*}$, if ${Q}^{*}>0.5$). Further, the data scatter is qualitatively similar for both types of RBCs, implying that there is no qualitative change in overall partitioning behavior at the network scale. However, the data points for different cell types are not overlapping, implying that differences exist in cell partitioning at the level of individual bifurcations.

To quantify the degree of alteration in time-average partitioning at individual bifurcations, we compute ${\Delta }_{N-Q}={({N}^{*}-{Q}^{*})}_{stiffer}-{({N}^{*}-{Q}^{*})}_{normal}$. The quantity $({N}^{*}-{Q}^{*})$ is a measure of degree of disproportionality in RBC partitioning; hence, ${\Delta }_{N-Q}$ provides a measure of change in disproportionality of RBC partitioning due to change in cell deformability. A positive value of ${\Delta }_{N-Q}$ means the partitioning becomes ‘more regular’; that is, a bifurcation exhibiting the regular partitioning for normal RBCs becomes more disproportionate in presence of stiffer RBCs, and a bifurcation with the reverse partitioning either switches to the regular partitioning or becomes closer to proportionate. In contrast, ${\Delta }_{N-Q}<0$ implies that the partitioning becomes ‘less regular’ or ‘more reverse’; that is, a bifurcation showing the regular partitioning in presence of normal cells moves closer to being proportionate or switches to the reverse partitioning for stiffer cells, while a bifurcation with the reverse partitioning becomes more disproportionate. Figure 4b shows that ${\Delta }_{N-Q}>0$ for about 65% bifurcations, $\sim$ 0 for 10%, and < 0 for 25% bifurcations, implying that for majority of the bifurcations the partitioning becomes ‘more regular’ in presence of stiffer cells. The figure also shows that the values of ${\Delta }_{N-Q}$ are comparable to ${({N}^{*}-{Q}^{*})}_{normal}$, implying that the amount of change in disproportionality is similar to the degree of disproportionality itself, and hence, is significant.

**Figure 5:**
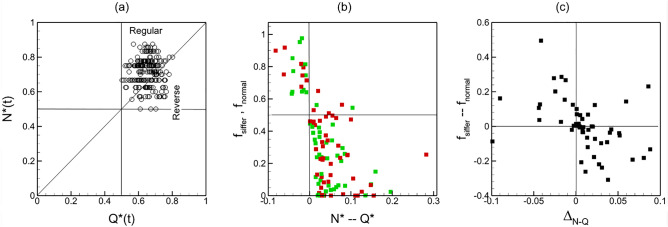
(**a**) Time-dependent partitioning at one selected bifurcation in a simulated network. Each data point represents an average over 0.1 s time window. (**b**) Fraction of time that the time-dependent partition is reverse at a bifurcation for normal (${f}_{normal}$, green symbols) and stiffer (${f}_{stiffer}$, red symbols) cells. (**c**) Effect of cell deformability on frequency of time-dependent reverse partitioning, computed as ${f}_{stiffer}-{f}_{normal}$, versus the change in time-average partitioning ${\Delta }_{N-Q}$.

The partitioning at any bifurcation is determined by the dynamics and interactions of the cells as they flow through the bifurcation. Because this is a highly dynamic process, it causes the partitioning behavior also to be time-dependent. Figure 5a shows the time-dependent partitioning plotted as ${N}^{*}(t)$ versus ${Q}^{*}(t)$ for one bifurcation. Each data point in this plot represents an average over an interval of 0.1 s. For this specific bifurcation, the time average partitioning is a regular partitioning. As seen, the time-dependent partitioning however fluctuates between the regular and reverse types. Such a fluctuating partitioning happens to be the case for most bifurcations, and for both normal and stiffer cells. Figure 5b shows the fraction of the total simulated time, ${f}_{normal}$ and ${f}_{stiffer}$, that the time-dependent partitioning becomes reverse for each bifurcation, for normal and stiffer cells, respectively. This fraction is simply the ratio of the number of data points below the ${N}^{*}={Q}^{*}$ line to that of the total number of points in the time-dependent partitioning plot, since the data points are sampled at equal intervals. As seen, for most bifurcations a time-dependent regular partitioning occurs more frequently if the time-average partitioning is also of the regular type ($f<0.5$ if ${N}^{*}-{Q}^{*}>0$), and vice versa ($f>0.5$ if ${N}^{*}-{Q}^{*}<0$). Exceptions are noted in a few bifurcations for which the time-average behavior is regular, but reverse partitioning occurs more frequently ($f>0.5$ if ${N}^{*}-{Q}^{*}>0$) . Interestingly, no bifurcation is observed to have a time-averaged reverse partitioning but frequent time-dependent regular partitioning ($f<0.5$ if ${N}^{*}-{Q}^{*}<0$).

Figure 5c shows the effect of cell deformability on frequency of time-dependent reverse partitioning, computed as ${f}_{stiffer}-{f}_{normal}$, versus the change in the time-average partitioning ${\Delta }_{N-Q}$. As seen, for most bifurcations, cell deformability affects the time-dependent and time-average partitioning in a similar manner. If the time-average partitioning becomes more regular (${\Delta }_{N-Q}>0$) in presence of stiffer cells, the frequency with which the time-dependent partitioning becomes reverse decreases (${f}_{stiffer}-{f}_{normal}<0$), and vice versa. Exceptions are noted for a few bifurcations for which the time-average partitioning can be more regular (${\Delta }_{N-Q}>0$), while the frequency of the time-dependent reverse partitioning also increases (${f}_{stiffer}-{f}_{normal}>0$).

**Figure 6:**
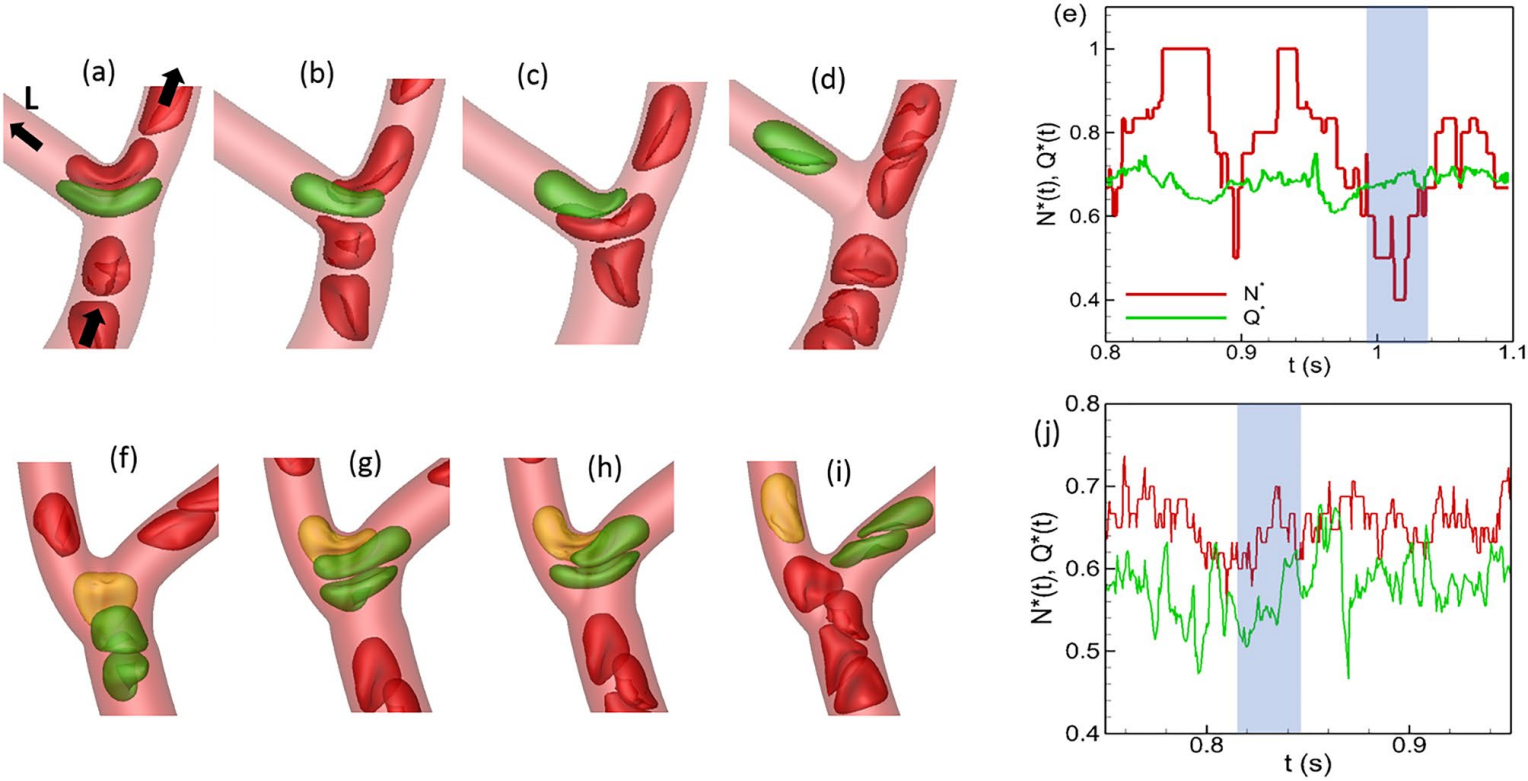
Alteration of time-dependent partitioning by cell lingering at a bifurcation. (**a**–**e**): A time-dependent reverse partitioning is caused by a cell momentarily blocking the higher flow rate branch of a bifurcation (right branch), causing a drop in ${N}^{*}(t)$ below ${Q}^{*}(t)$ in the time window marked by shading. (**f**–**j**) A lingering cell (yellow) blocking the lower flow rate branch (left branch) causing a time-dependent regular partitioning (shaded region).

We now seek to establish a link between the time-dependent partitioning alternating between regular and reverse types and the dynamics of individual RBCs as they flow through a bifurcation. For both normal and stiffer cells, one mechanism by which the partitioning behavior is altered is the cell ‘lingering’. Under this mechanism as an RBC approaches the apex of a bifurcation, it deforms significantly and straddles around the apex where the flow resembles a stagnation point/extensional flow^18,20–23^. The cell can remain at this location for some time as other cells flow around it. Depending on the geometry of the bifurcation, flow rates and hematocrits in the daughter vessels, and the shape of the lingering cell, various situations can arise as follows. The lingering cell can momentarily block the higher flow rate branch causing more cells upstream to enter the lower flow rate branch as shown in Fig. 6a–e. At times, the lingering cell itself can enter the lower flow rate branch if an increasing number of cells enter the higher flow rate branch raising the flow resistance and decreasing the flow rate. Both situations cause the lower flow rate branch to intermittently receive a higher fraction of cells, leading to a time-dependent reverse partitioning. Conversely, a lingering cell can block the entrance to the lower flow rate branch, thereby increasing the number of cells entering the higher flow rate branch and causing a time-dependent regular partitioning, as shown in Fig. 6f–j. Both of these opposite effects of lingering are observed for normal and stiffer cells. However, lingering causing a time-dependent reverse partitioning is generally seen to occur less in presence of stiffer cells, while lingering causing a time-dependent regular partitioning is observed to occur more.

**Figure 7:**
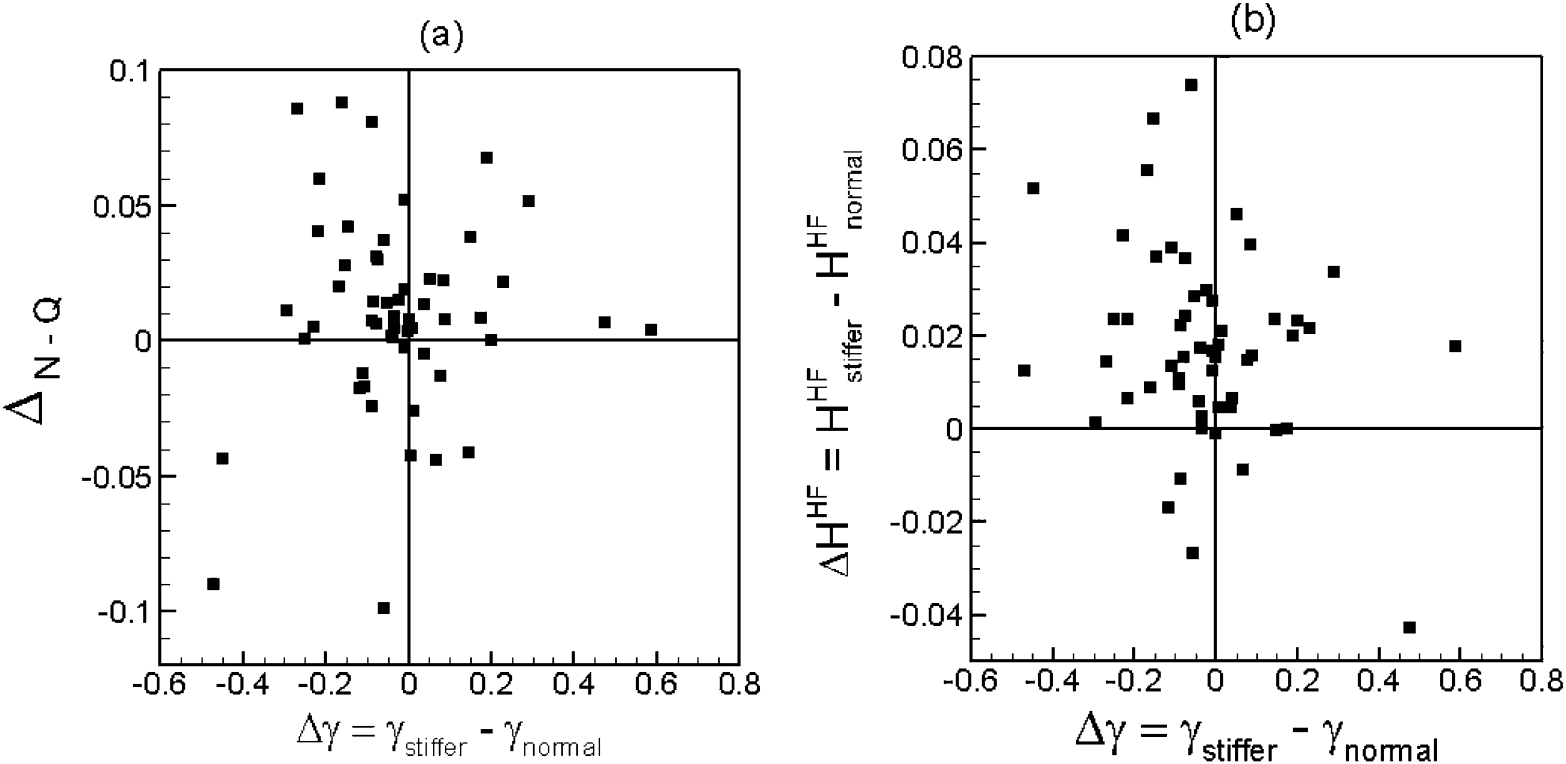
(**a**) Change in RBC partitioning ${\Delta }_{N-Q}$ versus change in the fraction of lingering RBCs $\Delta \gamma$. (**b**) Change in hematocrit ${\Delta {H}^{HF}={H}^{HF}}_{stiffer}-{{H}^{HF}}_{normal}$ in the higher flow rate daughter vessel of a bifurcation versus $\Delta \gamma$.

We now quantify the effect of RBC deformability on the lingering. From the simulation data, we compute the fraction of the total number of flowing cells at any bifurcation that linger, namely, ${\gamma }_{normal}$ and ${\gamma }_{stiffer}$, for normal and stiffer RBCs, respectively. The difference $\Delta \gamma ={\gamma }_{stiffer}-{\gamma }_{normal}$ gives the change in the fraction of lingering RBCs due to deformability change which is shown in Fig. 7a. As seen, $\Delta \gamma <0$ for more than half of the bifurcations, implying a reduction in the number of lingering cells as a result of reduced deformability. This generally happens at Y-shaped bifurcations where a more deformable cell as it approaches the bifurcation can significantly stretch so that two ends of the cell extend into both daughter vessels, before it can eventually enter one. This causes a greater number of deformable cells lingering at these bifurcations. As deformability decreases, RBCs do not significantly stretch thereby reducing the number of lingering cells at Y-shaped bifurcations. In contrast, for about 28% bifurcations $\Delta \gamma >0$, implying a greater number of cells lingering as deformability decreases. This second situation generally happens when a capillary vessel with a relatively smaller diameter comes off the side of a larger arteriole, and stiffer cells tend to get ‘stuck’ at the bifurcation due to the geometric constraint before eventually entering the side branch.

To investigate how the change in the lingering dynamics caused by RBC deformability affects the cell partitioning, we plot ${\Delta }_{N-Q}$ versus $\Delta \gamma$ in Fig. 7a. As seen, for about half of the bifurcations ${\Delta }_{N-Q}>0$ and $\Delta \gamma <0$, meaning that partitioning becomes more regular for the stiffer RBCs because of the reduced lingering events. That is, a reduced degree of lingering of the stiffer RBCs causes the higher flow rate branch to receive an even higher fraction of RBCs compared to the normal cells. Under the same mechanism, an increased lingering of stiffer cells can lead to more reverse partitioning (${\Delta }_{N-Q}<0$ and $\Delta \gamma >0$) which however happens for a very few bifurcations. This situation can arise when a stiffer lingering cell partly blocks the higher flow branch so that the RBC flux ratio to that branch is reduced more than the flow rate ratio in presence of stiffer cells. Conversely, for about 25% bifurcations, ${\Delta }_{N-Q}>0$ and $\Delta \gamma >0$, implying that partitioning becomes more regular due to increased lingering of stiffer cells. This situation is observed when a small capillary vessel comes off the side of a larger arteriole, as noted above. A stiffer lingering cell at such bifurcations can partly block the smaller side branch which allows no cell but only plasma to enter the side branch, causing the RBC flux ratio for the higher flow branch to increase more than the flow rate ratio.

Next, we seek to establish a link between the hematocrit change in individual vessel with the change in the lingering dynamics caused by RBC deformability. For this, we compute hematocrit change in the higher flow rate branch at any bifurcation, defined as ${\Delta {H}^{HF}={H}^{HF}}_{stiffer}-{{H}^{HF}}_{normal}$ , and consider its dependence on $\Delta \gamma$ as shown in Fig. 7b. For about half of the bifurcations, the data shows that $\Delta {H}^{HF}>0$ and $\Delta \gamma <0$, implying that the reduced degree of lingering of stiffer RBCs causes an increase in hematocrit in the higher flow rate branch. This mechanism is observed to be the dominant one in many vessels where large changes in hematocrit are predicted. Under the same mechanism, an increased degree of lingering of stiffer RBCs causes a decrease in hematocrit ($\Delta {H}^{HF}<0$ and $\Delta \gamma >0$) in the higher flow rate branch; this however happens only for a few bifurcations. For about 20% bifurcations, we predict $\Delta {H}^{HF}>0$ and $\Delta \gamma >0$, implying that a greater degree of lingering of stiffer RBCs causes an increase in hematocrit in the higher flow rate branch. As noted before, this situation occurs when a capillary vessel of small diameter emanates from the side of a larger arteriole, and a stiffer lingering cell blocks the passage of RBCs but allows plasma flow into the side branch.

This analysis shows that in most vessels the hematocrit change is directly caused by the change in the lingering dynamics as result of the loss of cell deformability which also causes the partitioning behavior to change.

### Additional mechanisms of hematocrit change

Apart from the cell lingering, two additional mechanisms are identified that affect partitioning behavior at a bifurcation and hematocrit in a daughter vessel, as discussed below.

**Figure 8:**
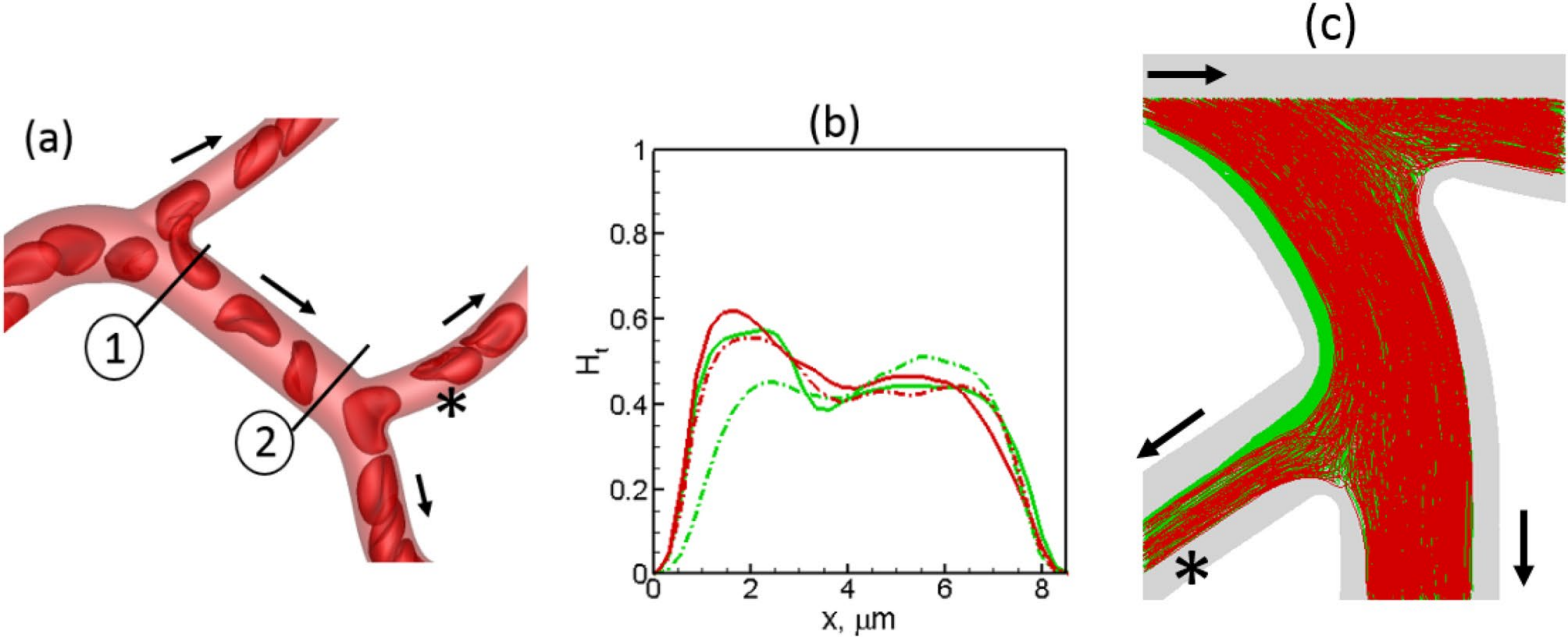
Additional mechanisms of hematocrit change. (**a**,**b**) Deformability causes a center-ward migration of RBCs, reducing the skewness of hematocrit profile more for normal cells than for stiffer cells. Hematocrit profiles at locations 1 (solid lines) and 2 (dash-dot lines) are shown in (**b**) for normal (green) and stiffer (red) RBCs. Nearly 30% increase in $H$ occurs by this mechanism in the vessel marked by*. (**c**) Vessel curvature effect. Trajectories of normal (green) and stiffer (red) RBCs are shown. Curvature effect causes a faster migration of more deformation cells toward the inner side of the vessel with the higher curvature. For the bifurcation selected here, this causes a smaller number of stiffer cells entering the vessel marked by *. Arrows indicate flow direction.

The first mechanism is related to the skewness of hematocrit distribution over the cross-section of a vessel, in conjunction with the deformability-induced cross-stream migration of cells, and is shown in Fig. 8a,b. Here the hematocrit profile at the beginning of the feeding vessel is skewed to the left side due to cell lingering at the upstream bifurcation. As the cells flow through the feeding vessel, the hematocrit skewness decreases because of center-ward cross-stream migration of deformable cells. This migration is faster for more deformable cells than stiffer cells^74^. As such, the hematocrit profile at the entrance to the downstream bifurcation remains more skewed toward the left daughter vessel for the stiffer cells compared to the normal cells. This leads to an increase of hematocrit ($\sim$ 30%) in this daughter vessel in presence of stiffer cells.

The second mechanism is related to vessel curvature effect. It was shown in our prior studies that deformable cells in a curved vessel migrate toward the side with higher curvature (i.e., the inner side), and the rate of such curvature-induced migration decreases with reduced deformability^67,75^. As a result, stiffer cells tend to flow further away from the inner side of a curved vessel than the normal cells. This can cause a hematocrit reduction for the stiffer cells in the daughter vessel that branches off from the inner side of the feeding vessel, as shown in Fig. 8c.

### Time-dependent behavior

**Figure 9:**
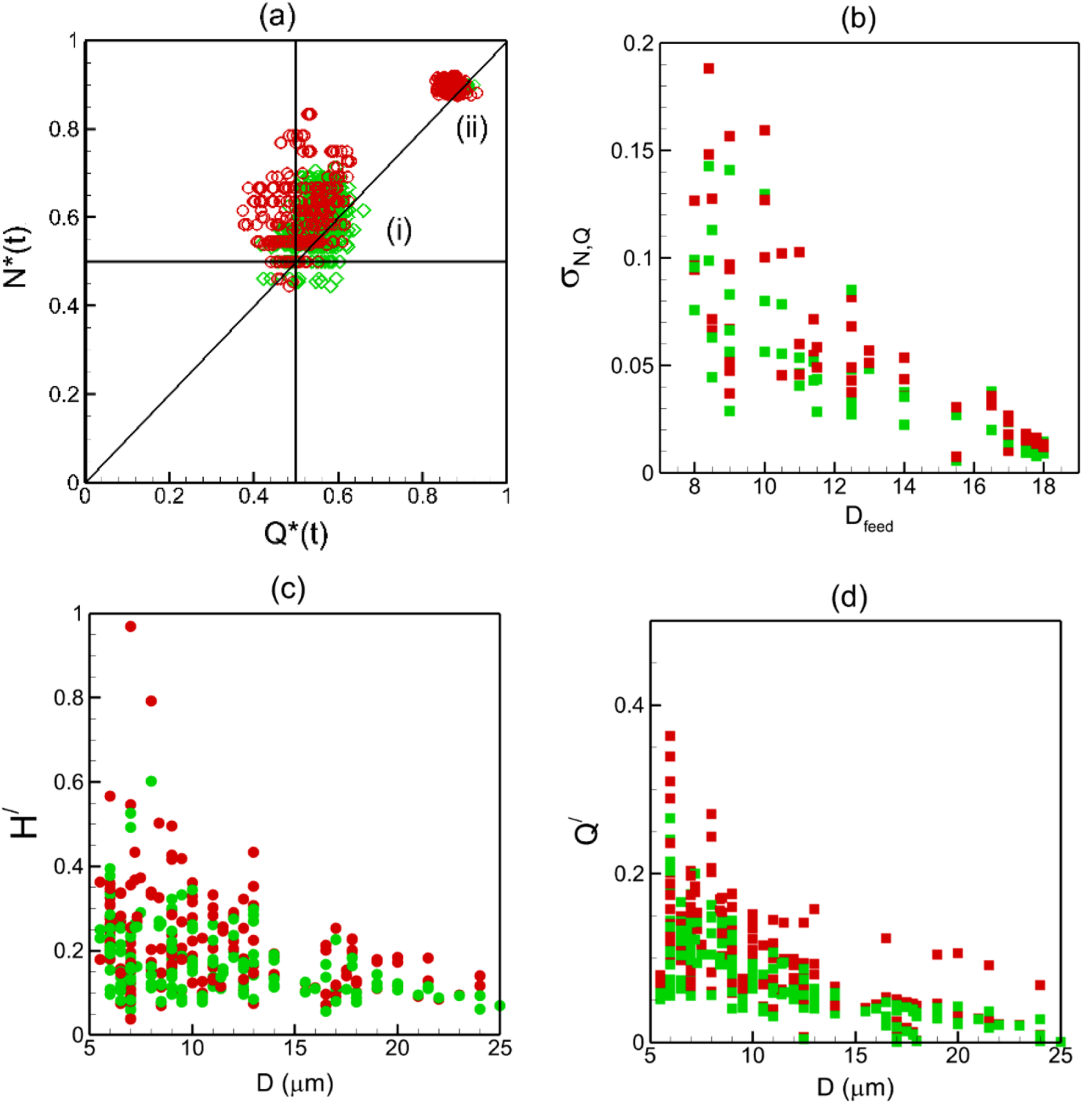
(**a**) Scatter of time-dependent partitioning for two different bifurcations: (i) smaller feeding capillary vessel (${D}_{feed}=8.5$ $\mu$ m) bifurcating to nearly similar daughter vessels (6.5 $\mu$ m) in Y-shape. (ii) larger feeder vessel (${D}_{feed}=17.5$ $\mu$ m) having a smaller side branch (6 $\mu$ m). Green and red indicate normal and stiffer cells, respectively. (**b**) Standard deviation ${\sigma }_{N,Q}$ of time-dependent partitioning w.r.t. the average as a function of feeder diameter. (**c**,**d**) Coefficient of variation of time-dependent hematocrit and flow rate, respectively, in each vessel as a function vessel diameter.

Alteration to the time-dependent partitioning due to reduced deformability also affects the flow and hematocrit variations in each vessel over time. As noted before, data scatter in ${N}^{*}(t)$—${Q}^{*}(t)$ plot represents the time-dependent partitioning. Figure 9a compares the time-dependent partitioning in two bifurcations with different feeding vessel diameters (${D}_{feed}$). Significant scatter is predicted for Y-shaped bifurcations for which the feeder vessels have relatively smaller diameters, and the two daughter vessels are of similar diameters. In contrast, the scatter is much less for a feeder vessel of larger diameter from which a smaller side branch emanates. This implies that partitioning undergoes a higher degree of fluctuations in time, and deviates more from the average for smaller feeder vessels than larger ones. This happens because a higher degree of cell lingering occurs for the former case than the latter. This trend is observed for both normal and stiffer cells. To quantify the degree of scatter in the time-dependent partitioning, we compute the standard deviation as ${\sigma }_{N,Q}=\sqrt{\frac{\sum_{i=1}^{M}\left\{{\left({N}^{*}(t)-{N}^{*}\right)}^{2}+{\left({Q}^{*}(t)-{Q}^{*}\right)}^{2}\right\}}{M}}$, where $M$ is the total number of data points in the ${N}^{*}(t)$—${Q}^{*}(t)$ plot. This quantity is plotted in Fig. 9b as a function of feeder vessel diameter for both cell types. As seen, ${\sigma }_{N,Q}$ decreases with increasing ${D}_{feed}$, consistent with the discussion above.

Furthermore, Fig. 9b shows that in most vessels, ${\sigma }_{N,Q}$ is predicted to be higher for the stiffer cells. At first this result may seem contradictory to the previous observation that the stiffer cells exhibit less lingering; however, these two are consistent. Because of reduced lingering of the stiffer cells and increased regular partitioning, a small increase in ${Q}^{*}$ in a daughter vessel can cause a larger change in ${N}^{*}$ since the cells would enter this vessel without lingering. Similarly, for a daughter vessel getting the smaller fraction of ${Q}^{*}$ and ${N}^{*}$, a small reduction in ${Q}^{*}$ would cause a larger reduction in ${N}^{*}$ for the stiffer cells than for the normal cells. Both scenarios are consistent also with the previous observation that the partitioning becomes more regular with the stiffer cells.

Such time-dependent partitioning affects the flow rate and hematocrit fluctuations in daughter vessels. We compute coefficients of variation of flow rate and hematocrit fluctuations (${Q}^{^{\prime}}$ and ${H}^{^{\prime}}$, respectively), and plot them in Fig. 9c,d as functions of vessel diameter, and for both cell types. We find that the trends of ${Q}^{^{\prime}}$ and ${H}^{^{\prime}}$ with respect to vessel diameter and cell deformability follow that of ${\sigma }_{N,Q}$; that is, fluctuations in flow rate and hematocrit increase with decreasing vessel diameter and cell deformability. Therefore, such fluctuations are consequence of the time-dependent partitioning and alteration of the lingering behavior at bifurcations.

### Perfusion

**Figure 10:**
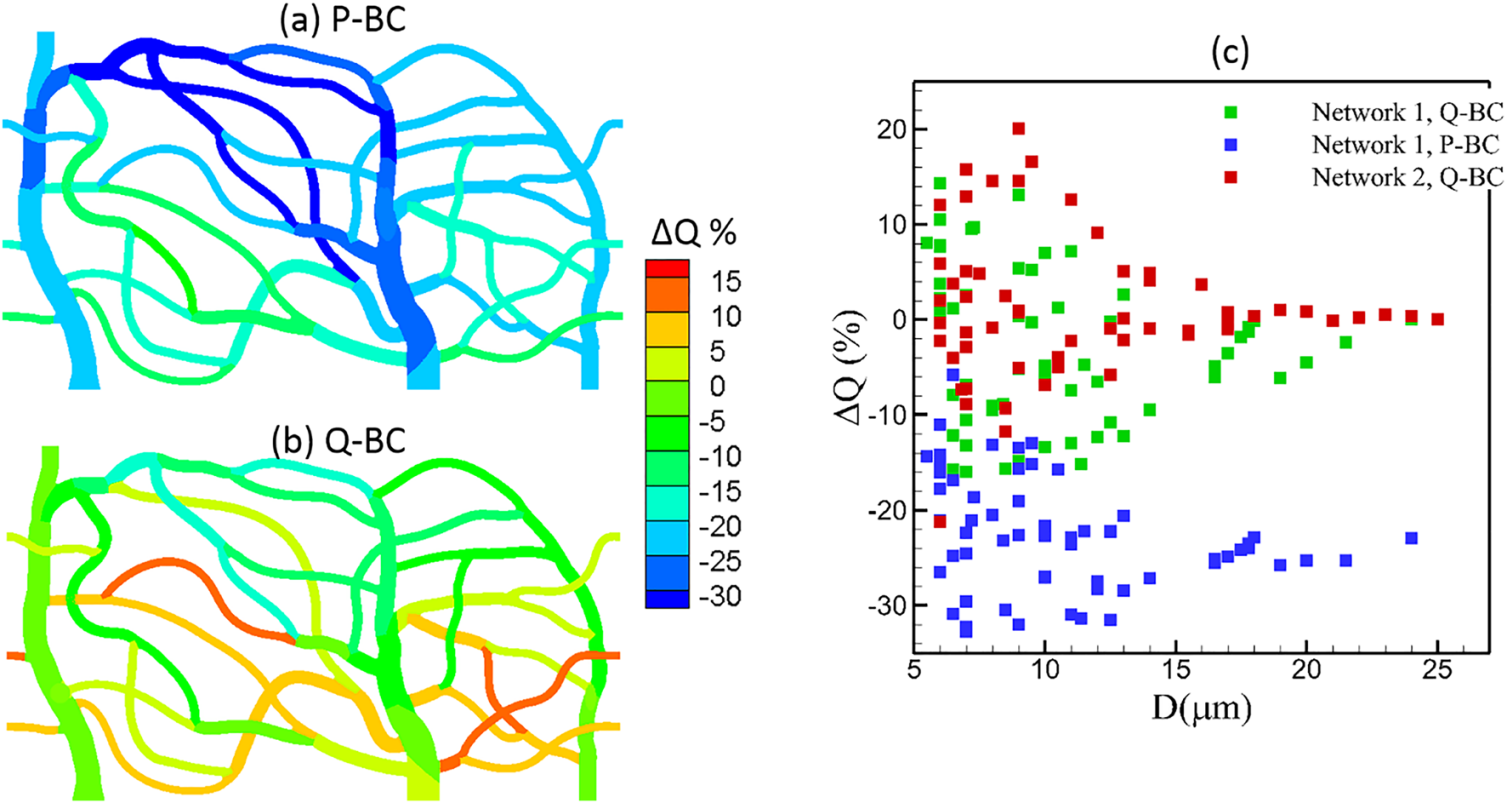
(**a**,**b**), Change is perfusion ΔQ = (Q_stiffer_—Q_normal_)/Q_normal_ in network 1 obtained from simulations with pressure (P-BC) and flow rate boundary conditions (Q-BC). (**c**) ΔQ as a function of vessel diameter from all simulations.

Next, we consider the time-average flow rate. Figure 10a,b shows maps of % change in vessel flow rate defined as ΔQ = (Q_stiffer_—Q_normal_)/Q_normal_ for network 1 obtained for simulations using flow rate boundary condition and pressure boundary condition. Figure 10c shows the data as a function of vessel diameter for both networks. For the pressure boundary condition, perfusion decreases in all vessels due to an increase in flow resistance caused by the stiffer cells. Although a reduction in perfusion throughout the network is observed, the change is heterogeneous, as can be seen from the map. The maximum decrease which is about 30% is observed in some capillary vessels located farthest from the inlet. For the flow rate boundary condition, the mean flow at the network scale does not change since the flow rate at the feeding artery is kept same for both cell types. However, individual vessels exhibit spatially heterogeneous changes, with some vessels showing a reduction in flow rate while others showing an increase. For both boundary conditions, the spatial heterogeneity in perfusion is higher in the vessels with smaller diameter.

**Figure 11:**
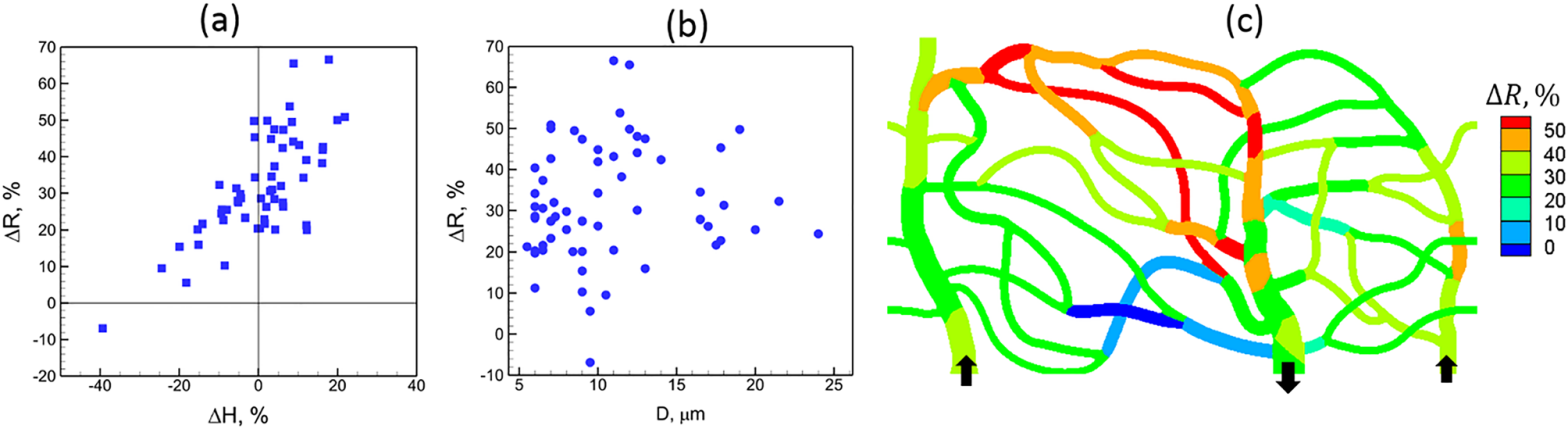
(**a**) Percentage change in flow resistance per vessel against percentage change in hematocrit. (**b**) Percentage change in flow resistance against vessel diameter. (**c**) Distribution of $\Delta R$ across the network.

Two possible mechanisms can cause the predicted heterogeneity in the perfusion change: (i) The alteration of RBC distribution due to the change in their deformability as predicted by our model can result in altering the flow resistance at individual vessel, and hence its perfusion; (ii) even if there is no alteration of the RBC distribution, the network-wide heterogeneity of hematocrit itself can cause different degree of changes in the flow resistance, and hence, vessel perfusion. For example, a vessel with a higher hematocrit would see a greater increase in the flow resistance due to the loss of RBC deformability than a vessel with a lower hematocrit. To further explore these two mechanisms, we plot % change in flow resistance ($\Delta R$) in each vessel against % change in hematocrit ($\Delta H$) in Fig. 11a. Predicted $\Delta R$ ranges from about ‒10% to 70% as $\Delta H$ varies from about ‒40% to 20%. As seen in the plot, when $\Delta H\sim 0$, $\Delta R$ is predicted to be about 20‒50%. This accounts for the effect of RBC deformability only. For nearly 1/3^rd^ of vessels, $\Delta R$ is less than this range due to a decrease in hematocrit, while a significant number of vessels exhibit higher $\Delta R$ due to an increase in hematocrit. Figure 11b presents $\Delta R$ as a function of vessel diameter. The scatter of the data implies a strong heterogeneity of $\Delta R$ across the network, with vessels of similar caliber showing large differences in $\Delta R$. Generally, the smaller capillaries show more heterogeneity, since the hematocrit change is also more heterogeneous in such vessels as shown previously. Figure 11c shows a spatial map of $\Delta R$ across the network. This illustrates geographically heterogeneous nature of $\Delta R$. Some of the peripheral capillary vessels exhibit the highest increase in the flow resistance. When compared to the map of $\Delta H$, a spatial correlation can be observed between the distribution of $\Delta R$ and $\Delta H$.

Generally, the flow resistance in a vessel, which can be expressed as effective blood viscosity, depends on vessel diameter, shear rate and hematocrit. It can also be related to the cell-free layer which is developed primarily due to center-ward hydrodynamic force acting on a cell and dispersion effect due to cell–cell interaction. The cell-free layer (CFL) near the wall provides a region of low resistance, while the RBC-rich region provides a higher resistance. In Refs.^81,82^ it is shown that for vessel diameter $d\approx$ 10—100 $\mu$ m, a vessel length of 25 $d$ is required to reach a converged CFL. Most vessels considered in the current in silico networks have lengths less than this, in agreement with in vivo data^10,83^. Furthermore, Ref.^81^ also noted that RBC dispersion effect would be small for $d\lesssim 20$ μm, which is also the case for most vessels in our networks. Additionally, most vessels are not straight. Therefore, CFL is not expected to be converged as shown also in our previous work^67^, and the flow resistance predicted here may be higher than a long straight vessel. In our simulations, we kept the hemoglobin to plasma viscosity ratio as 5 for both normal and stiffer cells. Ref.^82^ observed that the influence of hemoglobin viscosity in this range has only a weak effect on CFL and flow resistance.

### Wall shear stress

Next, we consider how the wall shear stress (WSS) is altered in the networks due to RBC deformability. Because of the unsteady nature of the RBC flow, the actual WSS varies in space and time. However, our focus here is on the time-averaged WSS. It is determined from the time-averaged velocity field by computing the traction vector ${\varvec{t}}={\varvec{\tau}}\bullet {\varvec{n}}$ at the wall, where ${\varvec{\tau}}$ is the stress tensor, and ${\varvec{n}}$ is the unit normal vector. We find that the axial component ${t}_{s}=\mu \partial {u}_{s}/\partial r$ of ${\varvec{t}}$ is the dominant one, and hence refer to it as the WSS and denote by $\tau$. Here $s$ and $r$ represent the axial and radial directions, respectively, at any location on a vessel surface. Due to the presence of the plasma layer in the vicinity of the vessel wall, the plasma viscosity (which is the same for stiffer and normal cells) is used in the above expression for ${t}_{s}$.

**Figure 12:**
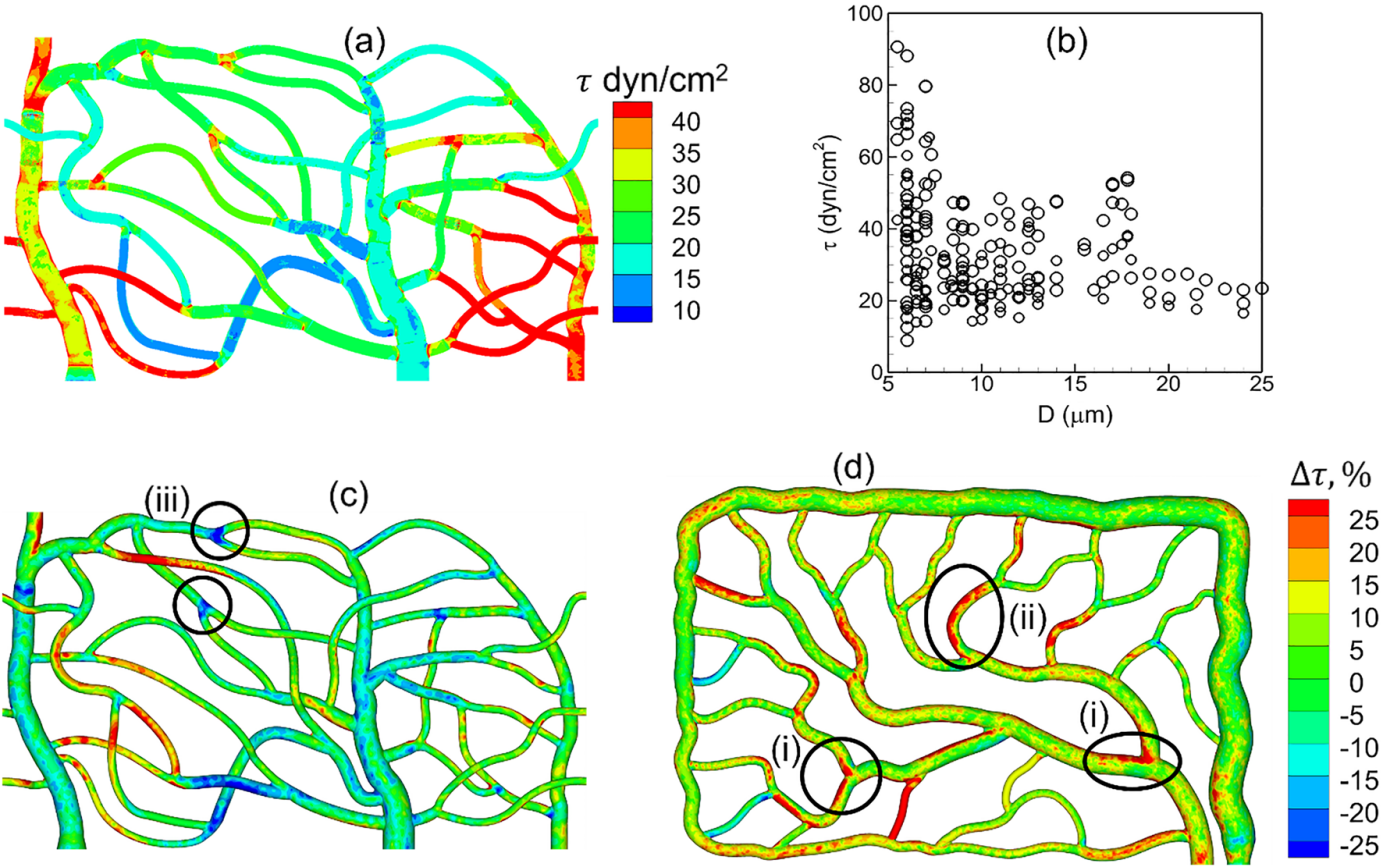
(**a**) 3D distribution of time-averaged WSS for normal RBCs. (**b**) Spatially averaged WSS per vessel in all simulations with normal RBCs. (**c**,**d**) relative change of WSS with stiffer cells. Regions showing local change in WSS are marked; (i) WSS increased at bifurcations, (ii) WSS increased in curved vessels, (iii) WSS decreased near bifurcations.

Because of the geometric complexity of the microvascular networks, the time-averaged WSS distribution is 3D in nature varying in both axial and circumferential directions, even within a single vessel. The complete 3D distribution is given in Fig. 12a as predicted for the network A for normal RBCs. In agreement with in vivo observations, WSS is seen to be higher in vessels on the arterial side than the venous side and is the highest in capillaries (see Ref.^66^ for comparison of predicted WSS with in vivo data). Additionally, WSS is predicted to be higher at capillary bifurcations. Further, a wide variability in WSS is observed from one vessel to another within the same group. Though the in vivo measurements and theoretical predictions based on 1D networks models of capillary blood flow provide a constant value of WSS per vessel^62,76,77^, our results reveal that WSS within a vessel has a strong 3D spatial variation which causes large spatial gradients in axial and circumferential directions. We also compute spatially averaged WSS for each vessel as $\iint \tau dA/A$ where $A$ is the vascular surface area. This is presented in Fig. 12b as a function of vessel diameter for all simulations with normal RBCs. The predicted WSS in capillary vessels range from about 10 to 90 dyn/cm^2^ which agrees with in vivo data^76,77^. Also, WSS increases with decreasing diameter, in agreement with in vivo observations and theoretical prediction, but unlike Murray’s law^62,66,76,77^. Similar variability of WSS and 3D distribution are observed when stiffer RBCs are considered.

We now consider the influence of cell deformability on WSS alteration. The relative change in WSS in presence of stiffer cells, defined as $\Delta \tau =({\tau }_{stiffer}-{\tau }_{normal})/{\tau }_{normal}$, is presented in Fig. 12c,d. As seen, $\Delta \tau$ is in the range $\pm 25\%$. These changes are highly localized in some vessels or in regions within a specific vessel where they can vary from large positive to negative values. No definitive correlation is found between $\Delta \tau$ and $\tau$. However, three generic patterns can be inferred from the spatial distribution of $\Delta \tau$ as follows: (i) A large positive $\Delta \tau$ may occur around the apex of a bifurcation, which can extend further downstream in a daughter vessel along the side that is closer to the apex. (ii) For a curved vessel, a large positive $\Delta \tau$ may also occur along the exterior side (i.e., the side with the higher radius of curvature). (iii) A negative $\Delta \tau$ occurs around capillary bifurcations in regions upstream and away from the apex. Next, we investigate the cellular mechanisms underlying such localized changes in WSS. It may be noted that because of such strongly varying $\Delta \tau$, spatially averaged $\Delta \tau$ over an entire vessel has a smaller range.

**Figure 13:**
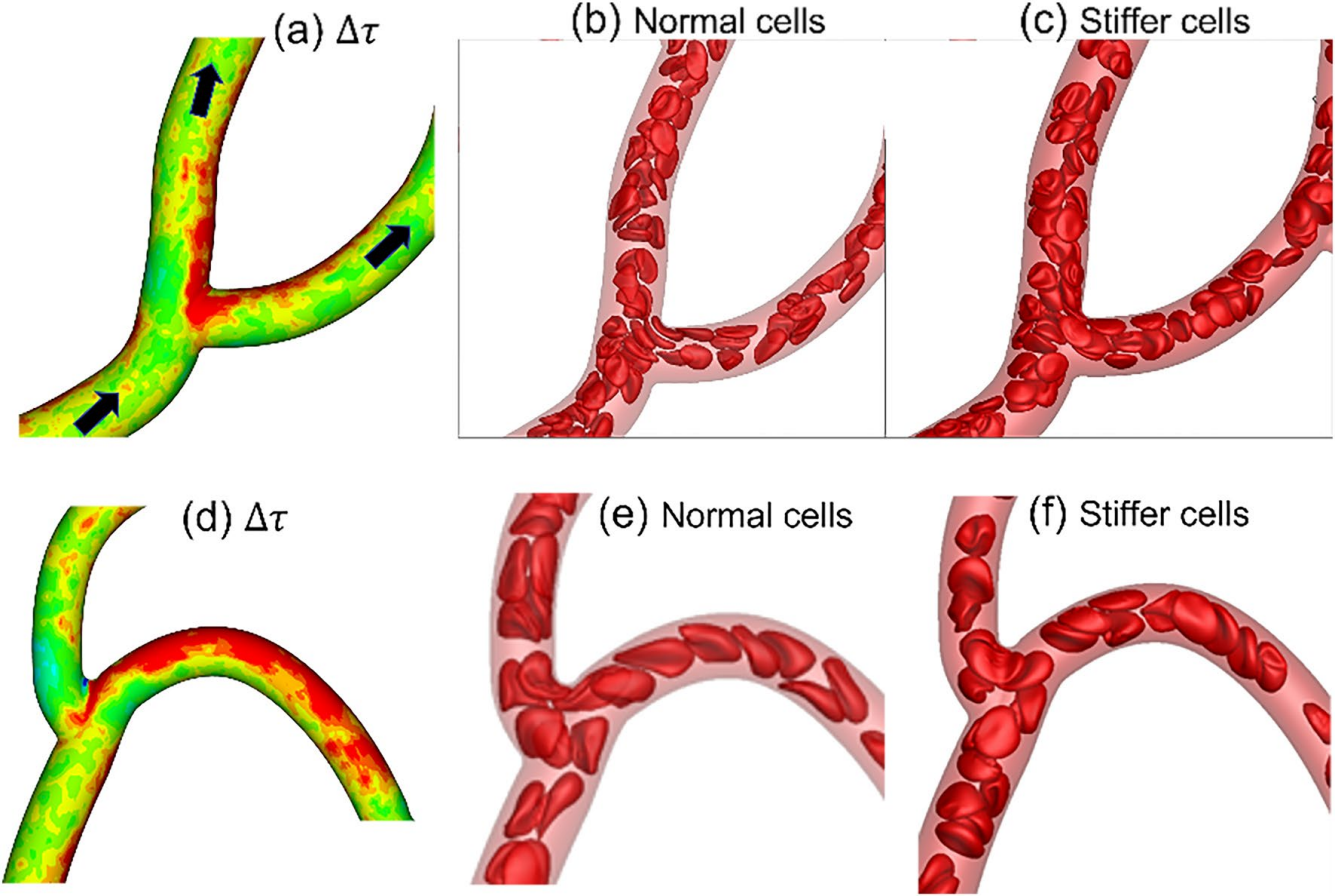
(**a**–**c**) Cellular-scale mechanism by which WSS increased near the apex of a bifurcation in presence of stiffer cells. (**d**–**f**) Mechanism by which WSS increased along the side of the vessel that has the higher radius of curvature. For (**a**,**d**) the color range is same as that in Fig. 12d.

The mechanism that causes the first of the above three patterns of localized large change in WSS is illustrated in Fig. 13a–c. If the average flow rate is kept fixed, the presence of RBCs can increase WSS in comparison to that in the presence of pure plasma flow by causing a blunt velocity profile. An increased proximity of the cells to the vessel wall causes a further increase in WSS by increasing the velocity gradient. For the bifurcations and vessels where the first mechanism is manifested, stiffer cells are observed to flow at closer proximity to the apex of the bifurcations and to the sides of the daughter vessels that are closer to the apex. Additionally, these bifurcations show a very small degree of lingering ($\gamma \lesssim 0.1$) for both types of cells. Together, these causes the velocity profile in presence of stiffer cells to be more skewed toward the side of the vessel that is closer to the apex, resulting in a higher WSS.

The mechanism by which a large positive $\Delta \tau$ appears in a curved vessel is illustrated in Fig. 13d–f. The vessel curvature causes a cross-streamline migration of deformable cells toward the interior side of the vessel (i.e., side with higher curvature) as discussed before. The migration rate decreases with a reduction of cell deformability. As a result, the stiffer cells tend to flow closer to the outer side of the vessel than the normal cells. This causes the velocity profile near the outer side to be more blunt, resulting a higher WSS with the stiffer cells.

The third pattern characterized by a large negative $\Delta \tau$ is observed at bifurcations where significant cell lingering is observed for both types of cells, and a higher degree of lingering for normal cells. Continued lingering causes a partial blockage around the apex displacing the freely flowing cells further toward the opposite walls, and causing a higher WSS in these regions. Since stiffer cells linger less at these bifurcations, this mechanism leads to a reduced WSS in these regions in presence of such cells.

## Discussion and conclusion

To predict blood flow in microvascular networks, previous theoretical models have often used 1D network flow models^61,62,69^. While such models allow consideration of large microvascular networks comprised of many blood vessels, they treat individual vessel as 1D segments. As such, these models cannot predict variation of hemodynamic parameters, such as velocity and hematocrit profiles over the vessel cross-section and variation of wall shear stress over the vessel surface and around vascular bifurcations. Such 3D variations cause local gradients in hemodynamic forces that can be sensed by the endothelial cells lining the vessel lumen and cause EC response that may be ‘focal’ in nature. Additionally, such 1D network models do not explicitly model deformation and flow of RBCs through the vessels, and therefore, may have limitations to study hemodynamic alteration caused by reduced RBC deformability as in sickle cell disease, diabetes, sepsis, and during transfusion of stored blood^27–38^. To overcome such limitations, here we have used a high-fidelity computational model of network blood flow that retains complete 3D geometric details of blood vessels and vascular bifurcations as observed in vivo, and accurately predicts 3D deformation of each of nearly a thousand flowing red blood cells. The in silico networks considered in our model span over tissue areas that are comparable to that of in vivo measurements in which multiple capillary vessels and flowing RBCs are simultaneously imaged^73^. Our ‘bottom-up’ approach allows varying the mechanical properties of individual cells and predicts the distribution and flow of the cells throughout the networks as develop naturally. The model predicts full 3D and time-dependent variations of hemodynamic parameters across the network and within individual vessels and connect them to behavior of individual cells with normal and abnormal properties.

Under normal healthy conditions, deformability of RBCs allows them to easily flow through vessels of diameter less than the cell diameter to ensure adequate tissue perfusion and oxygen delivery. RBC deformability is dictated by its hemorheological characteristics that include membrane viscoelasticity, hemoglobin viscosity and cell shape and surface are to volume ratio^11,27–38^. Depending on specific conditions, one or multiple of these parameters can change. While several studies have considered measuring such properties of diseased cells, the role of deformability on microvascular blood flow alteration is not completely established. Previous in vivo studies used chemically hardened cells or stored cells, and demonstrated loss of perfusion, which is generally explained by an increased blood viscosity^39–49^. Here we hypothesized that beyond such large-scale hemodynamic alteration there exists strong local changes that are directly linked to dynamics of individual RBCs. Our hypothesis is based on previous studies that reported focal alteration in microvascular network topology in aforementioned diseases, such as the appearance of microaneurysms, increased vessel tortuosity and capillary regression^3–5,59,60^. Studies that connect the alteration of individual RBC behavior due to reduced deformability with the local changes in hemodynamic parameters (e.g., 3D WSS) are lacking, which was the focus of this study.

One key finding of this study is that reduced RBC deformability significantly alters their trafficking as evidenced by the predicted change in hematocrit distribution. Microvascular distribution of RBCs is known to be spatially heterogeneous^10,14–17,24^, which is correctly predicted by our model. The coefficient of variation (CV) of the spatial heterogeneity predicted by our model ($\sim 29$%) is in the range reported in a previous in vivo study^16^. Although we predicted CV increased by a small amount ($\sim 17$%) when stiffer cells are considered, hematocrit change at the level of individual capillary was predicted to be significant. While some capillaries show a large increase in hematocrit, others show a decrease. The change in hematocrit is also spatially heterogeneous with the terminal capillaries generally showing the greatest change. The increased heterogeneity due to reduced deformability predicted here agrees with in vitro studies of RBC flow in artificial microfluidic networks^43^, as well as in vivo studies with parasitized RBCs in malaria^30^.

We then investigated the cellular-scale mechanisms that cause large hematocrit changes in individual vessels, and found that for majority of vessels such changes are caused by the behavior of individual RBCs at the upstream bifurcations. Previous studies have demonstrated that when RBCs flow through a bifurcation, they generally partition in a disproportionate manner in the daughter vessels downstream^10,12,14,17–26^. In most situations, the vessel receiving a higher fraction of the flow receives an even higher fraction of cells. In some bifurcations, the opposite behavior occurs. The former is termed as the regular partitioning, and the latter as the reverse partitioning. Here we found that for majority of vessels, reduced RBC deformability augmented the regular partitioning and attenuated the degree of reverse partitioning.

We established a link between altered cell partitioning and dynamics of individual RBCs at a bifurcation. Previous studies by other investigators and our works have demonstrated that the transit of an RBC may slow down near the apex of a bifurcation where a stagnation flow exists^18,20–23^. The cell can linger at this location for some time and stretch on both sides of the apex before eventually entering one of the daughter vessels. The lingering cell can temporarily block the higher flow branch, thereby directing the upstream cells into the lower flow branch and causing a time-dependent reverse partitioning. We found that reduced deformability resulted in a reduction of the number of lingering cells in most bifurcations. Therefore, the higher flow branches receive an even higher fraction of RBCs compared to the normal cells. This causes a hematocrit increase in the higher flow rate branch in presence of stiffer cells.

The above mechanism is, however, not the only mechanism by which hematocrit change occurs for the stiffer cells. Since the in silico networks considered here contains many asymmetric bifurcations and winding vessels, other mechanisms also dictate hematocrit changes. For example, we found that a stiffer cell can partly block the higher flow branch in a bifurcation in a way such that the RBC flux ratio to that branch is reduced more than the flow rate ratio, thereby attenuating the degree of regular partitioning and causing a decrease in hematocrit in the higher flow branch, as opposed to what is noted above for majority of bifurcations. Conversely, a stiffer cell can partly block the lower flow branch causing the RBC flux ratio for the higher flow branch to increase more than the flow rate ratio, thereby augmenting the regular partitioning and causing an increase in hematocrit in the higher flow branch. In both situations, the stiffer cells linger more than the normal cells, unlike what was noted above for majority of bifurcations.

Additional mechanisms related to RBC dynamics that alter partitioning and vessel hematocrit are also found. One mechanism involves the cross-stream migration of RBCs and how that affects hematocrit skewness. Generally, the hematocrit distribution over the cross-section of a vessel is not axisymmetric, but rather skewed to one side^17,19,23–36^. This may be caused by the partitioning of cells at an upstream bifurcation where they tend to flow along the sides of the vessels that are closer to the bifurcation apex. It is well known that RBCs being highly flexible migrate across the flow streamline toward the center of the vessel^74^. Such cross-stream migration reduces the hematocrit skewness. The rate of migration decreases with reduced cell deformability, resulting a higher skewness for stiffer cells, and an increase in hematocrit in the daughter vessel of a downstream bifurcation that is favored by such skewed hematocrit profile. Additionally, we found that vessel curvature also affects partitioning. We previously reported that in a curved vessel, deformable cells migrate cross-stream toward the side of the vessel with higher curvature^67,75^. The rate of such curvature-induced migration also decreases with reduced deformability. As such, partitioning of normal cells may favor the branch that is closer to the higher curvature side while the reverse is the case for stiffer cells.

We further showed that due to reduced lingering, the time-dependent partitioning fluctuates more for the stiffer cells. This causes a higher degree of temporal oscillations in flow rate and hematocrit in presence of stiffer cells, as also reported previously in a study using microfluidic networks^43^. One possible implication of this result is that the spatial and temporal heterogeneity are not separate but coupled.

Spatially heterogeneous changes are also predicted for time-averaged flow rate and vessel resistance, with the peripheral vessels showing a greater amount of heterogeneity. A strong correlation is observed between change in resistance and that in hematocrit, implying that hematocrit redistribution due to reduced deformability plays a significant role in altering vessel perfusion. Such heterogeneous changes have physiological implications as they can trigger vascular adaptation to meet tissue metabolic demand^69^. Sustained reduction in vessel perfusion may also lead to vessel regression^59,60^. Our results therefore suggest that RBC rheology may contribute to morphological changes in capillary networks as observed in diabetic retinopathy and sickle cell vasculopathy^3–5^. This connection between cell dynamics and vascular geometry dictating cell partitioning is also predicted in a recent computational study that modeled 3D flow of cell suspension through Y-shaped bifurcations with part of the feeder vessel compressed^84^. There it was shown that upstream vessel compression results in focusing of RBCs, thereby altering their partitioning behavior.

Our model predicted strong 3D spatial variation of WSS over a network as well as within a vessel, unlike in previous in vivo data and theoretical prediction using 1D network models which predicted constant WSS per vessel^61,62,76,77^. This further enabled us to predict highly focal changes in WSS within each vessel as well as in bifurcation regions caused by reduced RBC deformability. We showed that WSS can either increase or decrease in a bifurcation depending on how RBCs are flowing through it. The former is the case in absence of a significant linger, while the latter is the case when normal cells linger more than stiffer cells. Additionally, a reduced cross-stream migration of stiffer cells in a curved vessel is shown to cause higher WSS on the side of the vessel with lower curvature. These results are also physiologically significant since WSS is known to trigger EC response. Furthermore, such focal changes in WSS caused by stiffer cells lead to large changes in WSS spatial gradient which is also known to trigger EC response^78,79^. It may be noted that high-resolution tomography images of large-vessel systems often yield a noisy reconstruction of the vascular surface and pose a problem for predicting WSS. As shown in Ref.^80^, the image should be smoothed to remove to avoid WSS artifacts which could be more severe near bifurcations. The current in silico networks, however, are not built in this way. The vessels are smooth, and do not pose such challenges.

Taken together, our results show how geometric complexity and RBCs dynamics simultaneously induce local changes in microvascular hemodynamics as a result of reduced RBC deformability. Although a specific RBC disease condition is not considered here, the computational model can be used for such purposes, such as sickle cell disease, diabetes mellitus and stored cells, each of which is characterized by an alteration in RBC mechanical properties. Additionally, vascular malformation and adaptation that are often associated with these and other disease conditions can be incorporated in the model allowing to simultaneously study the coupling of red blood cells’ altered rheology and alteration in vascular topology in disease initiation and progression.

One important issue is how the predicted results is affected as many aspects of the heterogeneity of the networks simulated here that depend on the distribution of vessel diameters, vessel lengths, branching ratios, network topology, etc. Because of this we have been very careful in building our in silico networks. First: To incorporate the heterogeneity to the extent possible, we build our models based directly on in vivo images and data on vessel diameters, lengths, branching angles, and topology. Second: to the extent allowed for reasonable computation times, we include as large tissue area as possible. This allows us to consider large numbers of vessels, bifurcations, and mergers (for the current paper, we have in total about 120 vessels, 42 bifurcations, and 42 mergers). Third: we further confirm that the distribution of diameter, lengths etc. follows the average distribution over the vessel generation/order as observed in vivo (e.g., discussed in^10^), e.g., Horton’s law for capillary diameter distribution over vessel generation (see^22^, Supplementary Materials). We are confident that these three considerations remove any “artificial heterogeneity” caused by the in silico geometry. Instead, the heterogeneity in hemodynamic quantities that is predicted by our model are “natural” as they agree with in vivo measurement as noted above. Further, the hemodynamic quantities predicted have been validated against in vivo measurements (e.g.,^22^, and the Supplement therein). Therefore, we believe that the conclusions drawn here are generic. As a further support to our point, the two networks considered here are from retinal and mesenteric microvasculature are topologically different. Yet, the predicted hemodynamic data (e.g., in Figs. 2–4, 10 etc.) are in the similar range. This further suggests that the results are generic. Furthermore, the heterogeneity of microvascular network topology varies from organ to organ (e.g., kidney glomerulus versus mesenteric versus cerebral) and under disease conditions (normal versus tumor). It would be very valuable to establish relationship between such topological heterogeneity and the resulting hemodynamic heterogeneity. While such issues can be addressed by our model, this is left for future studies.

The second issue is whether the current large-scale network model should be used to address microscale phenomena (e.g., RBC partitioning etc.), or such phenomena is better addressed in detail by “small-scale” geometry, e.g., single bifurcation. We note that n realistic physiological scenario, vessels are geometrically complex (e.g., winding) and multiple bifurcations and mergers occur sequentially. On one hand, such globally “connected” geometry affects the microscale phenomena; while on the other hand, the “affected” microscale phenomena further alter the global hemodynamics. The present approach allows us to study evolution of hemodynamic quantities in a “natural” way as they evolve in physiological conditions. We believe such advantages are not there in “smaller-scale” models such as one or two bifurcations in isolations.

It may be noted, as evident from our results, that the difference between normal and stiffer RBCs is manifested more in some specific hemodynamic quantities, but relatively less in others. For cell partitioning, it is known that even fully rigid spherical particles exhibit disproportionate partitioning. Thus, it is not surprising that stiffer RBCs exhibit disproportionate partitioning as so normal cells as predicted here. Further, the aggregate data—considering all bifurcations of networks appear to look relatively unchanged. But changes may be significant at the level of individual vessel. For other quantities, e.g., cell deformation, time-dependent fluctuations and perfusion, significant difference can be noted. We think that the origin of relatively small change in some vessels is as follows: The stiffer RBCs as considered here are still deformable, unlike hardened RBCs as considered in vivo/in vitro. The cells are able to squeeze through the smallest capillary, and there is no vessel where flow completely stopped. We think this is due to the same biconcave shape that provides the excess area needed for deformation of even the stiffer cells, as evident from Fig. 1a. As the cells are able to flow smoothly through all vessels, the distribution of flow and RBCs is naturally adjusted throughout the network so that perfusion is maintained.

Another important issue is how the current results can be used to develop correlations or phenomenological models that can allow the results to be applicable to different conditions. For this, there are two levels of complexity involved in the current approach—the geometric complexity of physiologically realistic microvascular networks and the fully resolved deformation of each cell out of nearly a thousand flowing. The heterogeneity of the network in terms of vessel diameter, length, branching angle and topology also poses another challenge. One needs a large number of such in silico networks to build some correlations, which is beyond the scope of this study. However, the conclusions derived in the present study should remain valid because they are drawn using known properties of cells (normal and stiffer) and physiological network geometry. Developing correlations or useful phenomenological models for different RBC disease conditions, such as sickle cell or malaria, or different microvascular abnormalities such as vasculopathy, would require each of such conditions to be considered separately and generate data for diverse networks topology using disease-specific RBC properties.

In conclusion, using a high-fidelity, 3D computational model that considers deformation of each RBC flowing through physiologically realistic microvascular networks, we investigated hemodynamic alteration due to reduced RBC deformability. We connected such hemodynamic changes to individual RBC dynamics. We showed that RBC trafficking is significantly but heterogeneously altered as a result of primarily the alteration of their behavior at vascular bifurcations, as well as in their cross-stream migration. Stiffer cells tend to linger less at majority of bifurcations augmenting the regular partitioning and attenuating the reverse partitioning. Changes in vascular resistance also correlate with hematocrit changes. Furthermore, alteration in RBC dynamics causes localized changes in WSS within vessel and in bifurcations.
